# *DUX4* reduction and muscle function improvement by subcutaneous delivery of gapmer antisense oligonucleotides

**DOI:** 10.1016/j.omtn.2025.102791

**Published:** 2025-12-08

**Authors:** Aiping Zhang, Kenji Rowel Q. Lim, Ze Chen, Toshifumi Yokota, Yi-Wen Chen

**Affiliations:** 1 Center for Precision Medicine and Genomic Research, Children’s National Hospital, Washington, DC 20010, USA; 2 Department of Medical Genetics, Faculty of Medicine and Dentistry, University of Alberta, Edmonton, AB T6G2H7, Canada; 3 Muscular Dystrophy Canada Research Chair, Edmonton, AB T6G2H7, Canada; 4 Department of Pediatrics, School of Medicine and Health Science, George Washington University, Washington, DC 20052, USA; 5 Department of Biochemistry and Molecular Medicine, School of Medicine and Health Science, George Washington University, Washington, DC 20052, USA

**Keywords:** MT: Oligonucleotides: therapies and applications, FSHD, *DUX4*, antisense oligonucleotide, gapmer, *D4Z4*

## Abstract

Facioscapulohumeral muscular dystrophy (FSHD) is caused by aberrant expression of double homeodomain protein 4 (*DUX4*). The disease has no effective treatment. Previously we demonstrated effective *DUX4* knockdown *in vitro* and *in vivo* using 2′-O-methoxyethyl (2′MOE) and locked nucleic acid (LNA) gapmer antisense oligonucleotides delivered via intramuscular injections. This study aimed to evaluate *in vivo* efficacy of the gapmers via systemic delivery using mouse models expressing *DUX4* at different levels. First, we injected the gapmers subcutaneously to *FLExDUX4* mice at 20 mg/kg twice a week for 10 weeks. Results showed significant reduction in *DUX4* mRNA and improved muscle function, assessed by grip strength. Muscle fibrosis and circulating TGFβ1 levels were significantly reduced, approaching baseline level. A dose-dependent *DUX4* reduction was observed in 2′MOE gapmer treated. In the *ACTA1-MCM;FLExDUX4* model, where DUX4 expression was induced by tamoxifen (5 mg/kg), treatment with 2′MOE gapmers effectively reduced *DUX4*, improved muscle function, and decreased inflammation. These findings highlight the therapeutic potential of gapmer-based *DUX4* reduction, leading to phenotypic improvement and restoration of muscle function in FSHD mouse models.

## Graphical abstract

**Figure:**
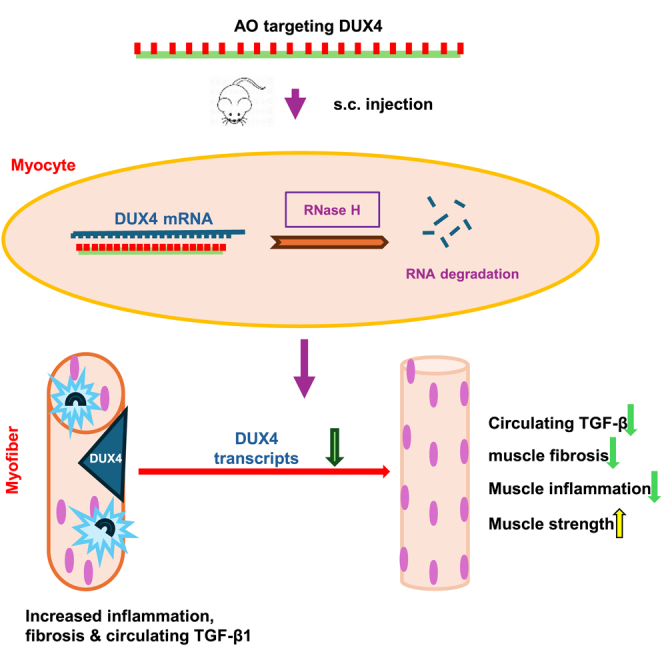

## Teaser

2′MOE-AO dose-dependently inhibits DUX4, and the dosage correlates to muscle uptake in FLExDUX4 mice. Long term subcutaneous administration significantly reduced DUX4 in several muscles of forelimb and hindlimb, reduced muscle pathology, and improved muscle function in FLExDUX4 and ACTA1-MCM;FLExDUX4 FSHD mice.

## Introduction

Facioscapulohumeral muscular dystrophy (FSHD) is an autosomal dominant skeletal muscle disorder, originally estimated to have a prevalence of approximately 1 in 20,000 individuals. However, later studies reported a higher prevalence of approximately 1 in 8,000, indicating a broader impact than initially recognized.^1^^,^^2^^,^^3^ FSHD is caused by complex genetic and epigenetic mechanisms, primarily involving the transcriptional de-repression of the *DUX4* gene located within the 3.3 kb macrosatellite repeat array, *D4Z4*, at the 4q35 subtelomeric region.^4^^,^^5^^,^^6^ Epigenetic changes in the *D4Z4* region are typically associated with the contraction of the *D4Z4* array from 11 to 150 repeat units in unaffected individuals to 1–10 repeat units in roughly 95% of patients with type 1 FSHD (FSHD1). In a smaller subset of patients with type 2 FSHD (FSHD2), mutations in the *SMCHD1*, *DNMT3B*, and *LRIF1* genes, which encode key epigenomic regulators, contribute to disease pathogenesis.^7^^,^^8^^,^^9^

Each repeat in the *D4Z4* region contains a *DUX4* open reading frame (ORF). Transcripts from the last *D4Z4* repeat are polyadenylated, which stabilizes the RNA for translation into the pathogenic *DUX4* protein. Under normal circumstances, *DUX4* is expressed in germ cells, particularly in the testis and four-cell embryos, but its expression is repressed in postnatal tissues, including skeletal muscle.^10^^,^^11^^,^^12^^,^^13^ Ectopic expression of *DUX4* has been shown to be embryonic lethal in various animal models, and it induces cell death in cells.^14^^,^^15^ While the downstream molecular changes associated with *DUX4* expression are recognized as the primary cause of FSHD, the precise mechanisms by which these changes lead to disease remain under active investigation.

Given its role in disease progression, strategies aimed at reducing *DUX4* expression hold promise as potential therapeutic approaches for FSHD. Various techniques have been explored to suppress pathogenic *DUX4* protein levels through the sequestration or degradation of its mRNA. These strategies include the use of small hairpin RNA (shRNA), microRNA (miRNA), small interfering RNA (siRNA), and antisense oligonucleotides (AOs).^7^^,^^16^^,^^17^^,^^18^^,^^19^ Although these strategies have shown considerable success *in vitro*, their vivo efficacy is often limited by factors such as poor delivery, stability, and specificity.

In this study, we utilized AOs to selectively reduce expression of *DUX4* mRNA. AOs are short, single-stranded DNA molecules typically ranging from 8 to 30 bases in length. They can be chemically modified to improve their stability, affinity for the target mRNA, and cellular uptake.^20^^,^^21^^,^^22^ The AO hybridized with their target transcript via Watson-Crick base pairing, resulting DNA/RNA duplexes, which are targeted for degradation by RNase H. To enhance the specificity, affinity, and uptake of AOs, we employed gapmer AOs, which incorporate modified nucleotides at both ends of the oligonucleotide to increase their efficacy.^23^^,^^24^ Specifically, we evaluated two types of gapmer AOs: one incorporating six locked nucleic acids (LNAs), three at each end, and the other incorporating ten 2′-O-methoxyethyl (2′MOE) nucleotides, five at each end. The chemistries (LNA and 2′MOE) have been extensively validated in both *in vitro* and *in vivo* settings.^25^^,^^26^^,^^27^^,^^28^ Previous studies conducted by our group have demonstrated efficacy of these gapmer AOs in cell cultures and in *FLExDUX4* mice via intramuscular injections.^27^^,^^28^ In this study, we evaluated one LNA and one 2′MOE gapmer AOs for further evaluation of *in vivo* efficacy via systemic delivery.

**Figure 1:**
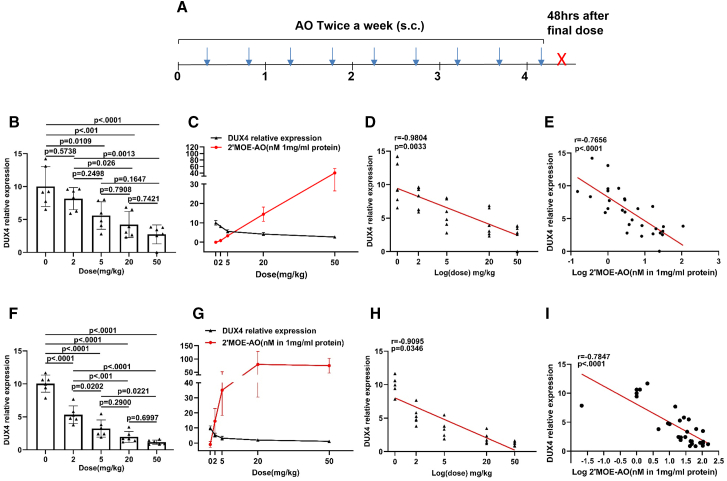
Dose-dependent inhibition of DUX4 and inverse correlation between DUX4 level and 2′MOE-AO muscle uptake in FLExDUX4 mice (A) Schematic of multidose treatment regimen. *FLExDUX4* mice received 2′MOE-AO (0, 2, 5, 20, or 50 mg/kg) subcutaneously twice weekly for nine doses (blue arrows) and were sacrificed 48 h after the final injection (red X). (B and F) *DUX4* expression level. (C and G) *DUX4* level and 2′MOE-AO muscle uptake curve are shown as a function of dosage (mg/kg). (D and H) Inverse correlation between DUX4 expression and log-transformed 2′MOE-AO dosage. (E and I) Inverse correlation between DUX4 expression and log-transformed 2′MOE-AO muscle concentration. All data are presented as mean (SD), *n* = 6 per group. Statistical significance was determined by one-way ANOVA with Tukey’s multiple comparisons test (*p* < 0.05). Correlations were assessed using Pearson’s correlation coefficient (r, *p* < 0.05).

In this study, both *FLExDUX4* and *ACTA1-MCM;FLExDUX4* models were used. The *FLExDUX4* mice spontaneously leak low levels of *DUX4*. We have shown these mice developed muscle fibrosis and muscle weakness.^29^^,^^30^^,^^31^ The *ACTA1-MCM;FLExDUX4* expresses *DUX4* at a higher level after tamoxifen-induction and developed more severe muscle phenotypes including muscle inflammation.^32^ A schematic illustration of the generation and genetic construction of these two models can be found in Figure 1 in the study by Takako et al.^32^ We reported dose-responses to the 2′MOE-AO gapmer, followed by efficacy of *DUX4* knockdown and phenotypic improvement in both the *FLExDUX4* and tamoxifen-induced *ACTA1.MCM;FLExDUX4* models. LNA-AO data suggested potential hepatic toxicity, therefore was only evaluated in one early trial using the *FLExDUX4* mice. The study highlights the therapeutic potential of gapmer-based *DUX4* reduction for treating FSHD.

## Results

### Dose-dependent reduction of *DUX4* in *FLExDUX4* mice treated with 2′MOE gapmer

To evaluate the dose-dependent efficacy of 2′MOE AOs (2′MOE-AO), we administered a range of dosages of 2′MOE-AO to *FLExDUX4* mice. Six mice (3 male, 3 female) per dosage group were included. Each group received subcutaneous injections (s.c.) of 0 mg/kg, 2 mg/kg, 5 mg/kg, 20 mg/kg, or 50 mg/kg twice a week for a total of 9 doses. Mice were sacrificed 48 h after the final dose (Figure 1A). Quadriceps and triceps from each mouse were used to analyze *DUX4* expression and tissue uptake of the 2′MOE-AO.

In the study, we observed significant reductions in *DUX4* expression at 5 mg/kg (quadriceps: *p* = 0.0109; triceps: *p* < 0.0001), 20 mg/kg (quadriceps: *p* < 0.001; triceps: *p* < 0.0001), and 50 mg/kg (both quadriceps and triceps: *p* < 0.0001) (Figures 1B and 1F). Relative mRNA levels of *DUX4* decreased to 44.0% and 67.7% at 5 mg/kg, 57.7% and 80.0% at 20 mg/kg, and 72.6% and 88.5% at 50 mg/kg in quadriceps and triceps, respectively (Figures 1B and 1F). Both one-way ANOVA *p* < 0.0001 for quadriceps and triceps (Figures 1B and 1F). Muscle tissue distribution analysis of 2′MOE-AO revealed concentrations of 3.37 nM, 14.5 nM, and 40.3 nM per mg/ml of quadriceps protein concentration for the 5 mg/kg, 20 mg/kg, and 50 mg/kg groups, respectively (Figure 1C). In triceps, corresponding concentrations were at 35.0 nM, 79.4 nM, and 74.9 nM per mg/ml of protein concentration (Figure 1G). Pairwise comparisons between dose groups showed no statistically significant differences in *DUX4* reduction between 5 mg/kg and 20 mg/kg, or between 20 mg/kg and 50 mg/kg, in either quadriceps or triceps.

The relationship between dose and the reduction of *DUX4* expression in both quadriceps and triceps was found to be highly correlated, with a Pearson correlation coefficient of r = −0.9804, *p* = 0.0033 (Figure 1D), r = −0.9095, *p* = 0.0346 (Figure 1H), respectively. Furthermore, the muscle uptake of 2′MOE-AO, expressed as log-transformed concentration (log[nM]), also showed a significant negative correlation with *DUX4* expression (r = −0.7656, *p* < 0.0001, Figure 1E; r = −0.7847, *p* < 0.0001; Figure 1I). The data show a significant association between the effectiveness of *DUX4* reduction and its retention within skeletal muscle. Notably, the 20 mg/kg dose group exhibited a robust reduction in *DUX4* expression despite relatively lower levels of muscle-retained 2′MOE-AO (Figures 1C and 1G), suggesting a favorable therapeutic window at this dose. Based on these findings, the 20 mg/kg dose was selected for all subsequent experiments in this study.

### Treatment of 2′MOE gapmer reduces *DUX4* expression and improves muscle pathology and function in the *FLExDUX4* mice

**Figure 2:**
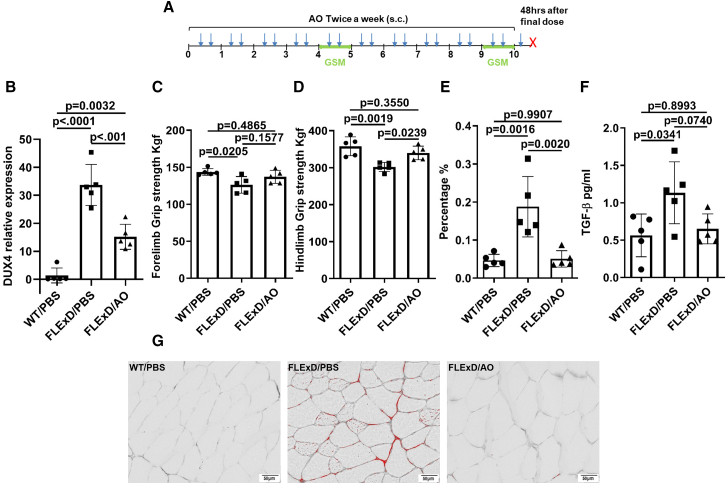
2′MOE-AO reduced *DUX4* level and improved muscle function and pathology in 10 weeks trial with *FLExDUX4* Mice (A) Schematic of the 10-week treatment regimen. FLExDUX4 mice received 2′MOE-AO (20 mg/kg) twice weekly via subcutaneous (s.c.) injection for a total of 21 doses (blue arrow). Grip strength measurements (GSM) were performed at week 5 (mid-point) and week 10 (endpoint). Mice were sacrificed 48 h after the final dose (red X). (B) *DUX4* expression in triceps muscle. (C and D) Forelimb (C) and hindlimb (D) grip strength measurements at the endpoint (week 10). (E) Percentage of fibrotic area relative to total muscle section. (F) Serum TGF-β levels. (G) Representative Picrosirius red staining images. Quantification was performed using ImageJ (green channel analysis). Scale bar, 50 μm. WT/PBS, wild-type littermates treated with the same volume of vehicle PBS, FLExD/PBS, *FLExDUX4* mice treated with vehicle PBS, FLExD/AO, *FLExDUX4* mice treated with 2′MOE-AO. All data are presented as mean (SD), *n* = 5 per group, one-way ANOVA with Tukey’s multiple comparisons test, *p* < 0.05 as significant.

We investigated the therapeutic potential of 2′MOE gapmer in a 10-week trial using the *FLExDUX4* mouse model, which leaks DUX4 at a very low level and develops endomysial fibrosis and muscle weakness.^29^^,^^31^^,^^32^ Six weeks old mice were administered 20 mg/kg of 2′MOE gapmer or diluent (PBS) via subcutaneous injections (s.c.) twice a week, for 10 weeks. Grip strength was assessed at two time points: week 5 and week 10 of treatment. Mice were sacrificed 48 h after the final injection (Figure 2A).

Our results show that *DUX4* mRNA level from triceps was reduced significantly (55.0%, *p* < 0.001) after 10 weeks of treatment (Figure 2B). Hindlimb strength by grip strength measurement (GSM) was significantly improved by the 2′MOE-AO treatment relative to the diluent control (*p* = 0.0239, Figure 2D). A similar trend was observed in the forelimb strength but did not reach significance (*p* = 0.1577, Figure 2C). Both forelimb (Figure 2C) and hindlimb (Figure 2D) strength in WT/PBS were higher than in FLExD/PBS and FLExD/AO (*p* = 0.0205, *p* = 0.0019, respectively). After the treatment, there was no significant difference in strength of either forelimb (Figure 2C) or hindlimb (Figure 2D) at endpoint in WT/PBS vs. in FLExD/AO. Grip strength measurement in mid-time point did not observe significant difference between 2′MOE-AO treated to PBS treated.

Muscle fibrosis was evaluated using Picrosirius red staining, which was used to quantify collagen density in muscle fibers. Our data showed a significant reduction in muscle of quadriceps fibrosis in the 2′MOE gapmer-treated group compared to PBS-treated controls (*p* = 0.0020, Figure 2E). Additionally, serum levels of activated TGF-β1, a key circulating biomarker of fibrosis, were lower in the 2′MOE gapmer-treated group (one-way ANOVA *p* = 0.0290, Tukey’s post hoc test, *p* = 0.0740; Figure 2F). Histological analysis (Figure 2G) revealed the presence of fibrosis localized to the endomysial region in affected muscles.

In this study, we did not observe significant differences in body weight or individual muscle weights between the treated and untreated groups throughout the study (Figures S1A and S1B). Furthermore, serum biochemistry analyses indicated no significant alterations in liver or kidney function in the treated mice (Figure S3A).

**Figure 3:**
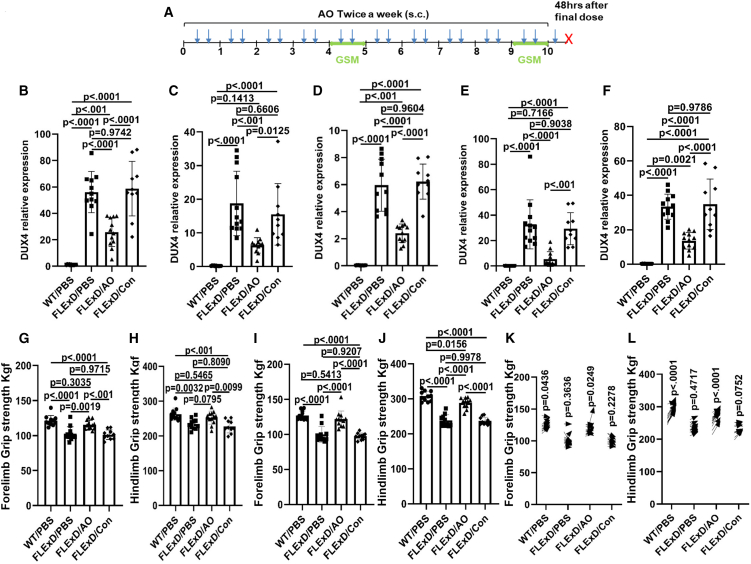
2′MOE-AO reduced *DUX4* and improved muscle function in repeat 10-week trial with *FLExDUX4* Mice (A) Schematic of the 10-week treatment regimen. FLExDUX4 mice received 2′MOE-AO (20 mg/kg) twice weekly via subcutaneous (s.c.) injection for a total of 21 doses (blue arrow). Grip strength measurements (GSM) were performed at week 5 (midpoint) and week 10 (endpoint). Mice were sacrificed 48 h after the final dose (red X). (B–F) DUX4 expression levels in (B) triceps, (C) biceps, (D) quadriceps, (E) tibialis anterior and (F) gastrocnemius muscles. (G–J) GSM of (G) forelimb and (H) hindlimb at the mid-point (week 5), and (I) forelimb and (J) hindlimb at the endpoint (week 10). (K and L) Comparison of GSM between mid-point and endpoint: (J) forelimb and (K) hindlimb. Arrows indicate the direction of change from mid-point to endpoint. WT/PBS, wild-type littermates treated with PBS (*n* = 11); FLExD/PBS, *FLExDUX4* mice treated with PBS (*n* = 11); FLExD/AO, *FLExDUX4* mice treated with 2′MOE-AO (*n* = 12); FLExD/Con, *FLExDUX4* mice treated with 2′MOE scramble control (*n* = 10). All data are presented as mean (SD), *p* value were calculated by using one-way ANOVA followed by Tukey’s multiple comparisons test for panels B–J, and two-way repeated-measures ANOVA with Tukey’s multiple comparisons test for panels K and L. p < 0.05 as significant.

To confirm that the observed effectiveness of 2′MOE-AO was not attributed to the 2′MOE chemistry itself, we repeated the study with higher sample size and added a mock 2′MOE-AO to the study. A total of 33 *FLExDUX4* mice (male, 6 weeks of age) were randomized into three treatment groups: *FLExDUX4* treated with vehicle (PBS) (FLExD/PBS); *FLExDUX4* treated with 2′MOE-AO (FLExD/AO), and *FLExDUX4* treated with the mock control oligonucleotide (FLExD/Con). The same dosing regimen of the gapmer AOs was administered over 10 weeks (Figure 3A).

Treatment with the 2′MOE gapmer resulted in a significant reduction of *DUX4* mRNA expression across multiple muscle groups, including triceps, biceps, quadriceps, tibialis anterior, and gastrocnemius. In triceps, *DUX4* levels were reduced by 53.9% and 55.9% relative to PBS- and mock-treated controls (both *p* < 0.0001, Figure 3B). In biceps, *DUX4* reductions of 66.5% and 59.4% were observed compared with PBS- and mock-treated groups (*p* < 0.001 and *p* = 0.0125, respectively; Figure 3C). Quadriceps showed *DUX4* decreases of 59.7% and 61.3% compared to PBS- and mock-treated controls (both *p* < 0.0001, Figure 3D). In tibialis anterior, *DUX4* expression was reduced by 83.6% and 81.6% compared to PBS- and mock-treated groups (*p* < 0.0001 and *p* < 0.001, respectively; Figure 3E). In gastrocnemius, *DUX4* expression was reduced by 59.4% and 60.7% compared to PBS- and mock-treated groups (both *p* < 0.0001; Figure 3F).

In contrast, no significant differences were observed between mock AO- and PBS-treated groups in triceps, biceps, quadriceps, tibialis anterior, or gastrocnemius. As expected, both the FLExD/PBS and FLExD/Con groups exhibited significantly elevated *DUX4* expression relative to the WT/PBS group across all muscle types examined (all *p* < 0.0001 for triceps, biceps, quadriceps, tibialis anterior, and gastrocnemius) (Figures 3B–3F).

Grip strength measurements revealed significant improvements in the 2′MOE gapmer-treated group. Specifically, forelimb grip strength at the mid-treatment time point was significantly greater in the 2′MOE gapmer group compared to either PBS or mock AO-treated groups (*p* = 0.0019 and *p* < 0.001, respectively; Figure 3G). Similarly, hindlimb grip strength was improved in the 2′MOE gapmer-treated group compared to either PBS or scramble-treated groups (*p* = 0.0795 and *p* = 0.0099, respectively; Figure 3H). At the mid-treatment time point, forelimb strength recovered to 94.8% to the wild-type levels, while hindlimb strength recovered to 95.8%. At the study endpoint, both forelimb and hindlimb strength remained significantly improved in the 2′MOE gapmer-treated group compared to the controls (both *p* < 0.0001, Figures 3I and 3J). Overall, forelimb and hindlimb strength recovery reached 96.7% and 94.3% of wild-type levels, respectively.

When comparing the grip strength data measured at the endpoint (week 10) to those collected at the midpoint (week 5), GSM data of forelimbs and hindlimbs significantly increased in the WT/PBS (*p* = 0.0436, *p* < 0.0001) and FLExD/AO (*p* = 0.0249, *p* < 0.0001) groups (Figures 3K and 3L). No significant increase of strength was observed in the FLExD/PBS or FLExD/Con groups (Figures 3K and 3L).

Serum biochemistry analysis of liver and kidney function panels revealed no evidence of significant toxicity in either the 2′MOE-AO or 2′MOE-Con treatment groups (Figure S3B).

### Treatment of LNA gapmer reduces *DUX4* expression and improves muscle pathology in the *FLExDUX4* mice

**Figure 4:**
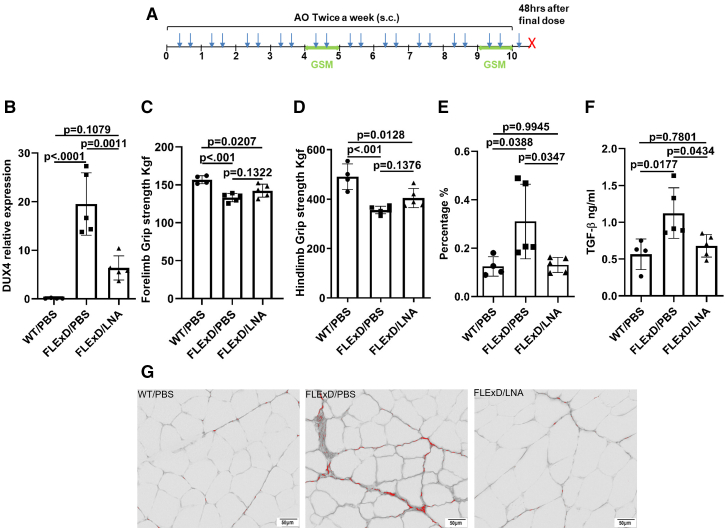
LNA-AO reduced *DUX4* level and improved muscle pathology in 10 weeks trial with *FLExDUX4* Mice (A) Schematic of the 10-week treatment regimen. FLExDUX4 mice received LNA-AO (20 mg/kg) twice weekly via subcutaneous (s.c.) injection for a total of 21 doses (blue arrow). Grip strength measurements (GSM) were performed at weeks 5 and 10. Mice were sacrificed 48 h after the final dose (red X). (B) DUX4 expression levels in quadriceps. (C and D) GSM of (C) forelimb and (D) hindlimb at the endpoint (week 10). (E) Percentage of fibrotic area relative to total muscle section. (F) Serum levels of TGF-β. (G) Representative Picrosirius red staining images of quadriceps muscle. Images were analyzed using ImageJ (green channel). Scale bar: 50 μm. WT/PBS, wild-type littermates treated with PBS (*n* = 4); FLExD/PBS, *FLExDUX4* mice treated with PBS (*n* = 5); FLExD/LNA, *FLExDUX4* mice treated with LNA-AO (*n* = 5). All data are presented as mean (SD), *p* value were calculated by one-way ANOVA with Tukey’s multiple comparisons test, *p* < 0.05 as significant.

In this experiment, we assessed the therapeutic efficacy of LNA gapmer in *FLExDUX4* mice. Mice received 20 mg/kg of LNA gapmer subcutaneously twice a week for 10 weeks. Wild-type littermates were used as controls. Grip strength was evaluated at two time points: week 5 and week 10 (Figure 4A). LNA gapmer treatment resulted in a significant 67.4% reduction in *DUX4* mRNA expression (quadriceps) compared to the FLExD/PBS control group, *p* = 0.0011 (Figure 4B), *DUX4* transcript levels were lower in WT/PBS than in FLExD/PBS but not significant difference in FLExD/LNA, *p* < 0.0001, *p* = 0.1079, respectively (Figure 4B). Expression of downstream *DUX4* target genes, Trim36 and Wfdc3, did not show significant changes (Figures S2C and S2D). One-way ANOVA revealed overall significant differences in forelimb and hindlimb grip strength (*p* = 0.0010 and *p* < 0.001, respectively; Figures 4C and 4D); however, post hoc analysis did not reach significance between groups.

Evaluation of muscle fibrosis through Picrosirius red staining revealed a significant reduction in fibrosis following LNA gapmer treatment, *p* = 0.0347 (Figure 4E). Additionally, serum levels of activated TGF-β1, a critical biomarker of fibrosis, were significantly reduced in the LNA-treated mice, *p* = 0.0434 (Figure 4F). Histological analysis (Figure 4G) revealed the presence of fibrosis localized to the endomysial region in affected muscles.

Throughout the treatment period, there were no significant differences in body weight between the LNA gapmer-treated and PBS-treated groups (Figure S2A). Similarly, no significant differences were observed in the weight of individual muscles between groups (Figure S2B). Biochemical analysis of serum samples revealed a marked increase in serum levels of alkaline phosphatase (ALP) and alanine aminotransferase (ALT) in the LNA-treated group (Figure S3A), suggesting potential liver or muscle-related effects. However, the clinical significance of these findings remains to be further clarified.

### Two-week treatment with 2′MOE-AO gapmer reduces *DUX4* levels and inflammation in *ACTA1-MCM;FLExDUX4* model

**Figure 5:**
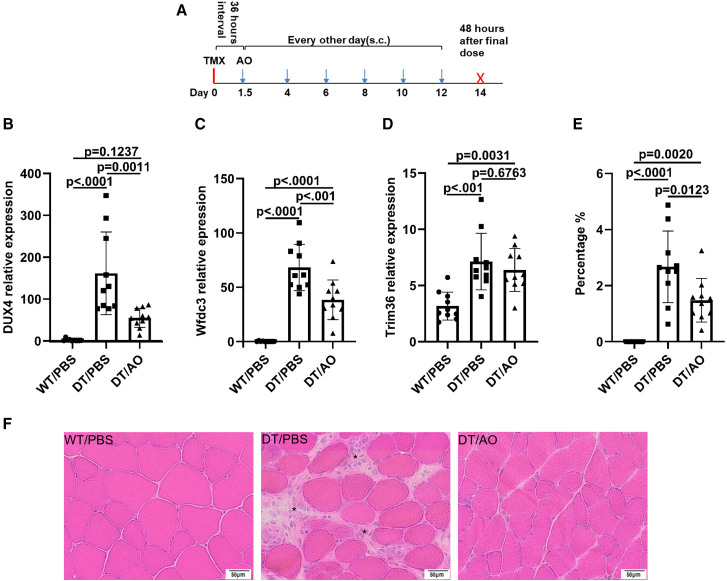
2′MOE-AO reduced *DUX4* level and muscle inflammation in short term trial with *ACTA1-MCM;FLExDUX4* mice (A) Schematic of the short-term treatment regimen. 2′MOE-AO (20 mg/kg) was administered every other day via subcutaneous (s.c.) injection for a total of six doses (blue arrow). Tamoxifen (5 mg/kg) was administered intraperitoneally (i.p.) 36 h before the first 2′MOE-AO dose (red line). Mice were sacrificed 48 h after the final 2′MOE-AO injection (red X). (B–D) Triceps expression levels of (B) *DUX4*, (C) *Wfdc3*, and (D) *Trim36*. (E) Percentage of inflammatory area relative to total muscle section. (F) Representative H&E staining of quadriceps muscle (scale bar, 50 μm). Asterisks ∗ indicate inflammatory foci. WT/PBS, ACTA1-MCM Cre-positive wild-type littermates treated with PBS; DT/PBS, ACTA1-MCM;FLExDUX4 mice treated with PBS; DT/AO, ACTA1-MCM;FLExDUX4 mice treated with 2′MOE-AO. All data are presented as mean (SD), *n* = 10 per group, *p* value were calculated by one-way ANOVA with Tukey’s multiple comparisons test, *p* < 0.05 as significant.

To evaluate the therapeutic efficacy of 2′MOE-AO gapmer in the *ACTA1-MCM;FLExDUX4* (DT) mouse model, we conducted a two-week treatment regimen. A total of twenty 6-week-old DT mice were randomized into two groups: 2′MOE-AO gapmer treatment and vehicle control (PBS), with *n* = 10 per group (5 males and 5 females). Wild-type littermates served as an additional control group and received PBS only. All mice were administered a single dose of tamoxifen (5 mg/kg, intraperitoneal) 36 h prior to the first 2′MOE-AO gapmer or PBS injection. The gapmer was administered subcutaneously at 20 mg/kg every other day for a total of six doses. Mice were euthanized 48 h after the final dose for tissue and serum collection (Figure 5A).

At the end of the treatment period, *DUX4* transcript levels in triceps were significantly reduced 66.5% in the 2′MOE-AO gapmer-treated mice compared to the PBS-treated group, *p* = 0.0011 (Figure 5B). Furthermore, the downstream target gene *Wfdc3* was significantly downregulated, *p* < 0.001 (Figure 5C), while the changes of expression of *Trim36*, another *DUX4* downstream target gene, did not reach significance, *p* = 0.6763 (Figure 5D).

Histopathological analysis through hematoxylin and eosin (H&E) staining revealed severe muscle fiber degeneration in untreated DT mice, with notable immune cell infiltration and inflammatory foci within the endomysium and perimysium (Figure 5F, DT/PBS). The 2′MOE-AO gapmer treatment resulted in improved muscle fiber integrity and a reduction in inflammation (Figure 5F, DT/AO). Quantification of inflammation foci using ImageJ confirmed a significant reduction in immune cell infiltration in the 2′MOE-AO gapmer-treated mice compared to the untreated DT mice, *p* = 0.0123 (Figure 5E). These findings indicate that 2′MOE-AO gapmer treatment not only reduces *DUX4* expression but also reduced muscle inflammation in the *ACTA1-MCM;FLExDUX4* model.

### Long-term efficacy of 2′MOE-AO treatment in improving muscle function and reducing inflammation in the *ACTA1-MCM;FLExDUX4* model

**Figure 6:**
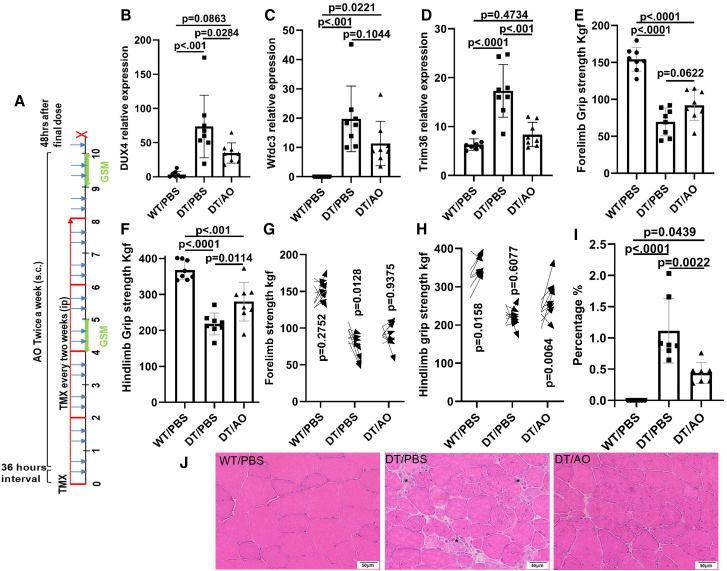
2′MOE-AO reduces DUX4 expression and inflammation and improves muscle function in long term trial with ACTA1-MCM;FLExDUX4 mice (A) Schematic of the treatment regimen. 2′MOE-AO (20 mg/kg) was administered twice weekly via subcutaneous (s.c.) injection for a total of 21 doses (blue arrow). Tamoxifen (5 mg/kg) was administered intraperitoneally (i.p.) every two weeks for a total of five injections (red line), 36 h before the first AO dose. Grip strength measurements (GSM) were performed at weeks 5 and 10. Mice were sacrificed 48 h after the final 2′MOE-AO dose (red X). (B–D) Triceps expression levels of (B) *DUX4*, (C) *Wfdc3*, and (D) *Trim36*. (E and F) GSM of (E) forelimb and (F) hindlimb at the endpoint (week 10). (G and H) Comparison of GSM between mid-point and endpoint: (G) forelimb and (H) hindlimb. Arrow direction indicates change from midpoint to endpoint. (I) Percentage of inflammatory area relative to total muscle section. (J) Representative H&E staining of quadriceps muscle. Scale bar, 50 μm; Asterisks ∗ indicate inflammatory foci. WT/PBS, *ACTA1-MCM Cre* positive and *DUX4* negative littermates treated with PBS; DT/PBS, *ACTA1.MCM;FLExDUX4* mice treated with PBS; DT/AO, *ACTA1-MCM;FLExDUX4* mice treated with 2′MOE-AO. All data are presented as mean (SD), *n* = 8 per group, *p* value were calculated by using one-way ANOVA followed by Tukey’s multiple comparisons test for panels B–F and I, and two-way repeated-measures ANOVA with Tukey’s multiple comparisons test for G and H. p < 0.05 as significant.

To evaluate the long-term efficacy of 2′MOE-AO gapmer in the *ACTA1-MCM;FLExDUX4* (DT) mouse model, a 10-week treatment study was conducted. Mice received 2′MOE-AO gapmer (20 mg/kg, s.c.) twice a week for 10 weeks. The first dose of 2′MOE-AO gapmer was administered 36 h after the first tamoxifen injection (5 mg/kg, intraperitoneal). To maintain *DUX4* expression, tamoxifen was administered every two weeks (5 mg/kg, intraperitoneal) according to previous study.^32^ Grip strength was measured at both the midpoint and endpoint of the study, with treatment beginning at 6 weeks of age (Figure 6A).

After 10 weeks of treatment, *DUX4* expression (triceps) was significantly reduced by 54.4% in the 2′MOE-AO-treated group, *p* = 0.0284 (Figure 6B). Interestingly, *Trim36* expression also decreased significantly, *p* < 0.001 (Figure 6D), while the changes of *Wfdc3* levels did not reach significance, *p* = 0.1044 (Figure 6C).

Functional assessments demonstrated significant improvements in both hindlimb and forelimb grip strength at the study endpoint in 2′MOE-AO treated mice compared with untreated DT mice (one-way ANOVA, both *p* < 0.0001). Post hoc analysis revealed a significant increase in hindlimb grip strength (*p* = 0.0114; Figure 6F), while forelimb strength showed an improvement that did not reach statistical significance (*p* = 0.0622; Figure 6E).

When comparing GSMs between the mid- and end-time points, no significant changes in forelimb strength were observed in the WT/PBS (*p* = 0.2752) or DT/AO (*p* = 0.9375) groups (Figure 6G), whereas a significant decrease was detected in the DT/PBS group (*p* = 0.0128, Figure 6G). The two-way ANOVA time factor (mid-time versus end-time) for forelimb GSM was not significant (*p* = 0.3898). In contrast, hindlimb GSM significantly increased from mid-time to end-time in both the WT/PBS (*p* = 0.0159) and FLExDUX4/AO (*p* = 0.0064) groups (Figure 6H), while no significant change was observed in the FLExDUX4/PBS group (*p* = 0.6077, Figure 6H). The ANOVA time factor (mid-time versus end-time) for hindlimb GSM was significant (*p* = 0.0074).

Histopathological analysis using hematoxylin and eosin (H&E) staining (Figure 6J) showed extensive endomysial inflammatory infiltration in untreated DT mice. In contrast, the 2′MOE-AO-treated group exhibited reduced immune cell infiltration. Quantification of inflammatory foci using ImageJ analysis confirmed a significant reduction in inflammation in the 2′MOE-AO-treated mice compared to untreated DT mice, *p* = 0.0022 (Figure 6I).

The body weights throughout the 10-week trial did not show significant differences between treated and untreated groups, although both groups exhibited lower body weights compared to the WT group (Figure S4A). Furthermore, no significant changes in weight were observed in individual skeletal muscles after treatment (Figure S4B).

Assessment of muscle fibrosis by Picrosirius red staining demonstrated no significant differences among groups, consistent with serum TGF-β1 measurements (Figures S4C and S4D). Western blot analysis of quadriceps muscle tissue detected faint DUX4-immunoreactive bands in the WT/PBS group. Although increased signal intensity was observed in the DT/PBS group, the antibody used lacked sufficient specificity to reliably differentiate DUX4 expression levels between groups (Figure S4E). This limitation underscores the need for more specific detection methods to accurately quantify DUX4 protein expression.

## Discussion

Facioscapulohumeral muscular dystrophy (FSHD) is a progressive skeletal muscle disorder for which no effective therapeutic interventions currently exist. Aberrant expression of the *DUX4* gene in the affected muscles is widely regarded as a key driver of FSHD pathogenesis, making gene therapy approaches, such as the use of AOs targeting *DUX4*, a promising therapeutic strategy.

Over the past two decades, considerable progress has been made in the development of AOs for treating FSHD. A comprehensive overview of published studies on AOs targeting *DUX4* is presented in Table S1. Various chemistries have been employed to enhance AO stability and efficacy, including phosphorodiamidate morpholino oligomers (PMO), 2′-O-methyl (2′-OMe), LNAs (LNA), 2′-O-methoxyethyl (2′MOE), constrained ethyl (cEt), and 2′-N-methanesulfonyl-2′-amino-locked nucleic acid (ALNA[MS]).^33^^,^^34^^,^^35^^,^^36^^,^^37^ Most studies have targeted Exon 3 and the polyadenylation signal (PAS) of *DUX4* mRNA (Table S1). Among these, five *in vivo* studies with *ACTA1-MCM;FLExDUX4* model have been reported, four of which used conjugated AOs (Table S2).

Lu-Nguyen et al.^33^^,^^34^^,^^35^ employed an octaguanidine dendrimer-conjugated PMO, achieving up to 60% reduction in *DUX4* expression. However, octaguanidinium dendrimer conjugation has been associated with potential cytotoxicity, hemolytic and hematological toxicity, reactive oxygen species generation, and pro-inflammatory responses.^38^^,^^39^ Moreover, its intraperitoneal delivery route is not clinically practical. Bouwman et al.^37^ used a cEt gapmer targeting exon 1, which achieved a 37% reduction in DUX4 expression (Table S2). Targeting exon 1 will also target *DUX4c* transcripts which may interfere with *DUX4c* function.^40^^,^^41^^,^^42^ Additionally, palmitoyl conjugation has been linked to neurological and cardiovascular side effects.^43^^,^^44^^,^^45^ Kakimoto et al.^36^ utilized an unconjugated ALNA[MS] gapmer with a phosphorothioate backbone, achieving approximately 35% *DUX4* reduction in the tibialis anterior muscle after 10-week treatment (10 mg/kg) and 45% reduction in the gastrocnemius after 6-week treatment (30 mg/kg). There was no evidence of liver or kidney toxicity, which suggested that ALNA[MS] gapmer can be a potential candidate for further drug development. In our study (Table S3), the unconjugated 2′MOE-AO targeting exon 3 achieved robust suppression of *DUX4* expression ranging from 53.9% to 83.6% across triceps, biceps, quadriceps, tibialis anterior, and gastrocnemius muscles (Table S3). In addition, the AO exhibited a favorable safety profile. Collectively, these data demonstrate that the unconjugated 2′MOE-AO gapmer is both highly effective and well-tolerated, underscoring its strong potential for clinical development.

In our study, we observed that the relative expression values of *DUX4* in different experiments were different. There are many factors that can contribute to the variations observed, including the fact that DUX4 expression in the *FLExDUX4* model is lower than that in the tamoxifen-induced *ACTA1-MCM;FLExDUX4* model. Repeated induction of DUX4 in the *ACTA1-MCM;FLExDUX4* was associated with lower *DUX4* expression. Additional factors include batches of reagents, different muscles, age, and sex of animals. Because the internal controls are only expected to be consistent in specific tissue as well as physiological conditions, we do not expect data from different muscles to be comparable. Therefore, all our samples were run in the same plate to allow proper comparisons. Since the values are relative expression values, we do not compare any data between plates.

Variability in grip strength measurements among trials was observed, which may be due to differences in mouse models (*FLExDUX4* vs. *ACTA1-MCM;FLExDUX4*), individual biological variations, and different batches of mice. Despite our efforts to minimize these factors by restricting body weight, age, sex and maintaining consistent experimental procedures, such variability could not be completely eliminated. Therefore, statistical comparisons were performed within each trial rather than across trials. Absolute GSM data were used instead of values normalized to body weight, as body weight gain in wild-type mice (or reduced gain in *FLExDUX4* and *ACTA1-MCM;FLExDUX4* mice) did not fully correlate with muscle mass or strength, and normalization could introduce bias.^46^^,^^47^ Nevertheless, both normalized and non-normalized data showed increased grip strength in treated mice.

Based on dose-ranging experiments evaluating efficacy and the distribution of 2′MOE-AO in the triceps and quadriceps muscles, a dose of 20 mg/kg was selected as the therapeutic window (Figures 1B, 1F, 1C, and 1G). Four independent therapeutic trials using this dose demonstrated significant reduction of *DUX4* expression and improvement in muscle function (Figures 2, 3, 5, and 6).

This is first time using the *FLExDUX4* mouse model to estimate drug efficacy with systemic delivery. In the trial significant reductions in *DUX4* transcripts were observed across five muscle groups: triceps, biceps, quadriceps, tibialis anterior, and gastrocnemius, following subcutaneous administration of 2′MOE-AO. These findings indicate that subcutaneous delivery of 2′MOE-AO effectively distributes to both forelimb and hindlimb muscles, supporting its potential for systemic treatment approaches *in vivo*.

When conducting Western blotting, although a band corresponding to the expected molecular weight of DUX4 (52 kDa) was observed in the positive control (from *DUX4* transfected myoblast lysate), the same band was also present in *Cre*-positive, *DUX4*-negative control mice. We could not clearly determine DUX4 protein level in *ACTA1-MCM;FLExDUX4* model (DT) (Figure 1, Figure 2, Figure 3, Figure 4, Figure 5, Figure 6E). While the DT/PBS group exhibited a stronger signal than both WT/PBS and DT/AO groups, the lack of specificity prevented definitive interpretation. To date, reliable quantification of low level DUX4 protein remains a significant challenge.

In parallel with the reduction in *DUX4* expression, significant improvements in muscle pathology and function were observed. After 10 weeks of 2′MOE gapmer treatment, *FLExDUX4* mice exhibited a 92% recovery of fibrosis, reaching levels comparable to wild-type mice, and a marked reduction in circulating TGF-β1 levels. These results further substantiate the therapeutic effect of 2′MOE gapmer in ameliorating muscle fibrosis, as evidenced by the reduction in TGF-β1, a well-established marker of fibrosis muscle diseases.^48^^,^^49^^,^^50^^,^^51^^,^^52^^,^^53^ Muscle grip strength also improved, reaching around 90% of the strength observed in wild-type mice (Figures 2C, 2D and 3G–3J). In the *ACTA1-MCM;FLExDUX4* model, immune cell infiltration into muscle fibers was reduced by 39.2% in the short-term trial (Figure 5E), and by 59.5% in the long-term trial (Figure 6I). Additionally, muscle grip strength in the long-term trial improved by 130% compared to untreated DT mice (Figures 6E and 6F). Interestingly, no significant fibrosis or elevated serum TGF-β1 levels were detected in the *ACTA1-MCM;FLExDUX4* model, warranting further investigation to clarify the underlying mechanisms and potential model-specific differences. Although individual skeletal muscle mass did not show a significant increase, overall muscle function improved, likely due to enhanced muscle quality resulting from reduced inflammation and fibrosis. Pharmacokinetic and dose-response data indicated a dose-dependent effect of the 2′MOE-AO gapmer, with a significant correlation between *DUX4* reduction and muscle uptake of the gapmer (Figure 2). These findings suggest that the 2′MOE-AO gapmer exhibits promising characteristics for further development as a therapeutic agent.

The LNA-AO and 2′MOE-AO gapmers used in the study targeted nearly the same site within exon 3 and demonstrated significant reductions in *DUX4* expression accompanied by improvements in muscle pathology. However, the LNA-AO has been reported to be associated with hepatotoxicity,^54^^,^^55^^,^^56^^,^^57^ which is supported by our experimental results. Given these safety concerns, further development of LNA gapmers is not warranted at this stage. In contrast, no toxicity has been reported to date for 2′MOE-AO in our 10-week trial; nevertheless, the potential for long-term adverse effects cannot be excluded and warrants further investigation.

Although *DUX4* expression in the *ACTA1-MCM;FLExDUX4* model is controllable, this model exhibits high *DUX4* levels and a severe phenotype.^32^ Myofibers expressing induced *DUX4* get replaced by regenerated myofibers that do not express induced *DUX4*. Maintaining levels of expression require repeated tamoxifen treatment every other week. In our study, we carefully timed regimen of tamoxifen administration in combination with the treatment of 2′MOE-AO gapmer. We were able to use this system to evaluate *DUX4* reduction and systemic therapeutic effects.

The *FLExDUX4* mouse model, characterized by low but detectable levels of *DUX4*-fl mRNA and mild phenotypic manifestations,^29^^,^^31^^,^^32^ presents a muscle fiber plasma membrane repair deficit,^30^ making it a valuable model for preclinical trials. This model avoids the complications associated with the severe muscle fiber regeneration cycles observed in model with induced *DUX4* expression, offering a useful platform for early-stage therapeutic evaluation.

In conclusion, our results show that the 2′MOE-AO gapmer is a promising therapeutic candidate for FSHD. The significant reduction in *DUX4* expression and subsequent improvement in muscle function observed in both the *FLExDUX4* and *ACTA1.MCM;FLExDUX4* models highlight the potential of this approach. Further investigations into the pharmacodynamics and toxicology studies of the 2′MOE-AO gapmer will be essential for advancing its development into a clinical treatment for FSHD.

## Materials and methods

### All animal procedures

All animal protocols were approved by the Institutional Animal Care and Use Committee (IACUC) of Children’s National Research Institute in Washington, DC. The FSHD-like mouse model used in this study consisted of *FLExDUX4* and *DUX4*-inducible *ACTA1-MCM;FLExDUX4* mice. The *FLExDUX4* model contains a floxed *DUX4* allele, which allows for conditional activation of the gene upon Cre recombinase-mediated excision of the loxP-flanked sequences. Mice in this model exhibit mild muscle weakness and slight pathology.^29^^,^^32^

For the *ACTA1-MCM;FLExDUX4* mice, crossing with the *ACTA1-MCM* mice allows the ACTA1 promoter to drive expression of the MCM protein, a tamoxifen-inducible Cre recombinase. This enables muscle-specific activation of the floxed *DUX4* gene upon tamoxifen administration.^29^^,^^32^ The dosage and timing of tamoxifen administration, as well as the timing of the AO treatment, were critical for the successful execution of the trial.

Subcutaneous injections at site of mouse back neck loose skin. If multiple doses, the injections intently avoid the same location of the skin. At the end of each experiment, mice were euthanized at the desired time points via CO2 inhalation followed by cervical dislocation. Blood was collected directly from the heart through an opened chest. Muscle samples, including quadriceps, gastrocnemius, tibialis anterior, triceps, and biceps, were surgically removed, flash-frozen in liquid nitrogen-chilled isopentane, and stored at −80°C for further analysis.

### Genotyping

*FLExDUX4* mouse genotyping was performed following the protocol developed by Peter L. Jones’s lab.^29^ The SurePlus Super Genotyping Kit (Amizona Scientific LLC) was used for the genotyping process. Only hemizygous mice were selected for inclusion in the study. Wild-type mice were confirmed to be negative for the *DUX4* allele.

For the *ACTA1-MCM;FLExDUX4* (DT) mouse genotyping,^29^^,^^32^ the same protocol was applied to confirm the presence of the *DUX4* transgene. Genotyping of the *ACTA1-MCM* allele was performed according to the Jackson lab’s protocol. The following primers were used for PCR amplification: Cre-F: 5′-AGG TGG ACC TGA TCA TGG AG-3′, Cre-R: 5′ ATA CCG GAG ATC ATG CAA GC-3′, internal positive control IPC-F: 5′-CTA GGC CAC AGA ATT GAA AGA TCT-3′, IPC-R: 5′-GTA GGT GGA AAT TCT AGC ATC ATC C-3’. Touchdown PCR was performed under the following conditions: 1. Initial denaturation 94°C 2 min; 2. denaturation: 94°C 20 s; 3. Annealing: 65°C 15 s, 4. Extension 68°C 10 s 5. Cycle repetition: repeated step 2 to 4 for 10 cycles, each cycle step 3 minus 0.5°C. 6. Final extension: 94°C 15 s, 60°C 15 s, 72°C 10 s, repeated for 28 cycles. 7. Final extension: 72°C 2 min, followed 10°C hold. PCR products were resolved on a 2% agarose gel prepared with TBE buffer (Thermo Fisher Scientific). The transgenic band was observed at approximately ∼440 bp. Mice confirmed to be positive because both *DUX4* and *Cre* were selected for inclusion in the AO (AO) trial. Wild-type control mice were selected based on a genotype positive for *Cre* but negative for *DUX4*.

### Antisense oligonucleotides design

In this experiment, two AOs were used: 2′-O-methoxyethyl AOs (2′MOE-AO) gapmer, 2′MOE-AO gapmer sequence is 5′CCUAGACAGCGTCGGAAGGU3’; 2′MOE scramble sequence is 5′AGCGCTGGCAAGGTATGCAC3′. Another AO modified by LNA which sequence is 5′CAGCGTCGGAAGGTG3′ (LNA-AO).

### Dosing experiment

A dose-dependent experiment was performed to assess the effects of varying dosages of 2′MOE-AO. Hemizygous 6-week-old male and female mice were randomly assigned to five groups, each receiving a different dosage of 2′-MOE-AO in PBS vehicle (150 μL). The dosage groups were: 0 mg/kg, 2 mg/kg, 5 mg/kg, 20 mg/kg, and 50 mg/kg. Mice in each group received subcutaneous injections twice a week for a total of 4 weeks, amounting to 9 injections in total. Body weight was recorded at each injection time point. Each group consisted of 6 mice (3 male and 3 female). Injections commenced at 6 weeks of age, and mice were euthanized 48 h following the final injection.

### Ten-week therapeutic experiment with *FLExDUX4* mice

*FLExDUX4* male mice were randomly assigned to two treatment groups: 2′MOE-AO treated, vehicle PBS treated, *n* = 5 per group. Another control group was its wild-type littermates (*n* = 5, male), with receiving vehicle PBS (150 μL). The treatment regimen involved 20 mg/kg of 2′MOE-AO or 2′MOE-scramble, administered subcutaneously twice a week for 10 weeks, totaling 21 injections. Injections began at 6 weeks of age. Body weight was recorded at each injection. Grip strength was assessed in week 5 and week 10. Mice were euthanized 48 h after the final injection, and muscle tissues and blood samples were collected. Muscle tissues were snap-frozen in liquid nitrogen-cooled isopentane and stored at −80°C for subsequent molecular analyses. Blood was collected via cardiac puncture, and serum samples were prepared, aliquoted, and stored at −80°C for later analyses.

For the repeated 2MOE-AO treatment on *FLExDUX4* mice*,* using the same design, but additional group for 2MOE-scramble control and increased sample size. 2′MOE-AO treated (*n* = 12, male), 2′MOE-scramble treated (*n* = 10, male), vehicle PBS treated (*n* = 11, male). Another control group was its wild-type littermates (*n* = 11, male), with receiving vehicle PBS (150 μL).

For LNA-AO trial on *FLExDUX4* mice, using the same design as above. Randomized groups were LNA-AO treated (*n* = 5, male), vehicle PBS treated (*n* = 5, male). Another control group was its wild-type littermates (*n* = 4, male), with receiving vehicle PBS (150 μL).

### Two-week therapeutic experiment with *ACTA1-MCM;FLExDUX4* mice

*ACTA1.MCM; FLExDUX4* double transgenic mice (DT) were randomized into two groups, 2′MOE-AO (*n* = 10, 5 female, 5 male), and vehicle PBS (*n* = 10, 5 female, 5 male). Another control group were littermates of *ACTA1-MCM;FLExDUX4* with negative DUX4 genotyping, and positive Cre genotyping (WT) (*n* = 10, 5 female, 5 male) with same volume vehicle PBS 150 μL. Regimen was 20 mg/kg, every other day, subcutaneous injection, total 6 doses. Before the first dose of 36 h, one dose tamoxifen (5 mg/kg) administrated by intraperitoneal injection. Mice started at 6 weeks of age. After the last treatment for 48 h, mice were euthanized, and muscle and blood sample were collected.

### Ten-week therapeutic experiment with *ACTA1-MCM;FLExDUX4* mice

*ACTA1.MCM; FLExDUX4* double transgenic (DT) male mice were randomized into two treatment groups: 2′MOE-AO treatment (*n* = 8), vehicle PBS treatment (*n* = 8). A separate wild-type (WT) control group was its littermates of *ACTA1-MCM;FLExDUX4* with negative *DUX4* genotyping, and positive Cre genotyping as a control group (*n* = 8), all treated with PBS vehicle. The experiment began when the mice were 6 weeks old. The 2′MOE-AO treatment regimen involved 20 mg/kg, administered subcutaneously twice a week for 10 weeks, with a total of 21 injections. The tamoxifen regimen was 5 mg/kg, administered intraperitoneally every two weeks for a total of 5 injections. The first dose of tamoxifen was administered 36 h before the initial dose of 2′MOE-AO treatment. Grip strength measurements were performed in week 5 and week 10 to assess muscle function.

After 48 h following the final dose of treatment, the mice were euthanized, and muscle tissues and blood were collected. Muscle samples were snap-frozen in liquid nitrogen-chilled isopentane and stored at −80°C for further molecular and histopathological analyses.

### Total RNA isolation and cDNA synthesis from muscle tissue

Frozen quadriceps, gastrocnemius, tibialis anterior, triceps, and biceps muscles were powdered in liquid nitrogen. The muscle powder was collected into a 1.5 mL eppendorf tube, and 100 mg sample was transferred to a new tube. To this, 1 mL of cooled TRIzol reagent (ThermoFisher Scientific, Cat# 15596026) was added for homogenization. Total RNA was isolated using the miRNeasy Micro Kit (Qiagen, Cat# 217084) following the manufacturer’s instructions. RNA concentration was measured using a NanoDrop One/OneC Microvolume UV-Vis Spectrophotometer (ThermoFisher, Cat# ND-ONEC-W). Between 0.5 and 2 μg of total RNA was used for cDNA synthesis using the SuperScript IV CellsDirect cDNA synthesis kit, according to the manufacturer’s instructions.

### Real-time quantitative RT-PCR

Real-time qRT-PCR was performed using a QuantStudio 7 Flex system (Life Technologies). Briefly, 20 ng of cDNA per sample was used for quantification. The following primers were used: ***DUX4***: *DUX4*-F 5′-CCCAGGTACCAGCAGACC-3′ (0.2 μM), *DUX4*-R 5′-TCCAGGAGATGTAACTCTAATCCA-3′ (0.2 μM); ***GAPDH***: GAPDH-F 5′-TTGTCAGCAATGCATCCTGC-3′ (0.2 μM), *GAPDH*-R 5′-CCGTTCAGCTCTGGGATGAC-3′ (0.2 μM), ***Trim36***: Trim36-F 5′-TGAAAGTGGGAGTTGCTTCC-3′ (0.2 μM), *Trim36*-R 5′-GAATCAAAACAGGCGTCCTC-3′ (0.2 μM); ***Wfdc3***: Wfdc3-F 5′-CTTCCATGTCAGGAGCTGTG-3′ (0.2 μM), *Wfdc3*-R 5′-ACCAGGATTCTGGGACATTG-3′ (0.2 μM). SYBR green PCR master mix (10 μL per sample, Applied Biosystems) was used, with a total reaction volume of 20 μL, adjusted by adding RNase- and DNase-free water. A negative control was included, which contained no sample but the reagent. Samples were amplified in triplicate using the following thermal cycling conditions. Initial denaturation at 95°C for 10 min, 40 cycles of amplification, denaturation at 95°C for 15 s, annealing/extension at 60°C for 1 min. The ΔΔCT method was used to determine the relative expression levels of genes normalized to GAPDH.

### Muscle cryosection

Cryosectioning was performed using a Leica CM 1950 cryostat (Leica Biosystems, Walldorf, Baden-Württemberg, Germany). The right quadriceps muscle was used for cryosection preparation. To prepare the cryosection block, the fresh or frozen quadriceps muscle was mounted on a cork with a gum mixture consisting of 6.8% tragacanth and 6 grains of thymol, ensuring the desired orientation. The mounted muscle was then immediately immersed in pre-chilled isopentane in liquid nitrogen for 1 min. After this, the cork with the mounted muscle was transferred to dry ice to evaporate the remaining isopentane before proceeding with cryosectioning. For H&E and Picrosirius red staining, cross-sections of the muscle were cut to a thickness of 8 μm at wide middle of quadriceps and mounted on Superfrost plus microscope slides.

### Hematoxylin and Eosin Staining

Hematoxylin and Eosin Staining (H&E) staining was performed following a standard protocol. Briefly, after slides were air-dried for 1 h at room temperature, they were incubated in hematoxylin (Hematoxylin 7211, Thermo Scientific) for 2 min, followed by rinsing in running water for 1 min. Next, slides were incubated in Clarifier 1 (Richard-Allan Scientific) for 1 min, then rinsed again in running water for 1 min. Following this, slides were incubated in Bluing Reagent (Richard-Allan Scientific) for 1 min, followed by another rinse in running water. Subsequently, slides were stained in eosin (Eosin Y, Richard-Allan Scientific) for 3 min to stain the cytoplasm. After staining, slides were dehydrated by sequential incubation in 95% ethanol for 1 min, repeated three times, followed by 100% ethanol for 1 min, repeated three times. The slides were then cleared in xylene for 1 min, repeated three times. Finally, coverslips were mounted using mounting oil, and the slides were prepared for microscopic scanning.

### Picrosirius Red staining

After cryosectioning, slides were air-dried for 1 h at room temperature. They were then incubated in xylene for 10 min, followed by rehydration through a series of ethanol washes: 100%, 95%, 80%, and 70% ethanol, each for 10 s. Next, slides were stained in 0.1% Picrosirius red (Sigma Aldrich, St. Louis, MO) for 60 min. After staining, the sections were incubated in 0.01N HCl for 2 min, followed by dehydration through a series of ethanol washes: 70%, 80%, 95%, and 100% ethanol, each for 10 s. Finally, slides were cleared in xylene for 5 min, twice, and mounted with coverslips.

### Microscope

All digital muscle images obtained through chemical staining were captured using the VS120 virtual slide microscope (Olympus America Inc.) set at 20× magnification to scan the entire tissue section.

### Fibrosis quantification in Picrosirius red staining

Digital images from entire tissue section were processed using ImageJ (http://rsb.info.nih.gov/ij), the green channel image applying the same threshold across all samples. The density corresponding to the red-stained area were quantified and normalized to the total section area. The results were expressed as the percentage of collagen accumulation.

### Inflammatory foci counting in H&E staining

Digital images from entire tissue section were used by counting inflammatory foci. The Inflammatory foci were blindly counted and their areas measured using ImageJ. The area of each inflammatory focus was quantified as a ratio of the focus area to the total section area. An inflammatory focus was defined as having seven or more infiltrating immune cells.

### Enzyme-linked immunosorbent assay

The concentration of active TGF-β1 in serum was measured using the TGF-β1 immunoassay system (R&D Systems, Minneapolis, MN). Serum samples were diluted 10-fold, and active TGF-β1 levels were quantified using an ELISA plate pre-coated with a specific TGF-β1 antibody, following the manufacturer’s protocol. Optical signal was measured at 450 nm within 30 min of stopping the reaction. A wavelength correction was performed by subtracting the value at 570 nm from the value at 450 nm.

### Grip strength measurement and calculation

Grip strength of the forelimb and hindlimb were measured using a Grip Strength Meter (Columbus Instruments) and measured at mid- and endpoint of each therapeutic trial. Briefly, the forelimb grid was positioned horizontally, while the hindlimb grid was angled. To assess grip strength, the mouse was gently held by the tail over the grid until it gripped the steel bars. The mouse was then pulled away from the grid, and the meter recorded the maximum force applied when the mouse released the bars. Before data collection, mice were acclimated to the device for five minutes each day for two consecutive days. All GSM were conducted by the same individual under blind conditions.

Grip strength measurement (GSM) was evaluated five gripping per one measurement for five consecutive days, and the average of the five grips for each day was recorded. The average grip strength over the five days was used as the final measurement for each mouse. These measurements were then analyzed to quantify grip strength.

### Blood biochemistry testing

A 150 μL serum sample from each mouse was sent to the MU Veterinary Medical Diagnostic Laboratory at the University of Missouri for serum liver and kidney biochemistry panel analysis. The test panel included measurements of serum urea nitrogen, creatinine, total bilirubin, ALP, ALT.

### Hybridization ligation enzyme linked immunosorbent assay

The same quadriceps and triceps samples collected for *DUX4* mRNA quantification were also used to assess 2′MOE-AO tissue uptake. The hybridization ligation enzyme-linked immunosorbent assay (HLELISA) was used for quantifying the uptake of 2′MOE-AO in target muscle. The template probe consists of a phosphodiester oligo-DNA that is complementary to the target gapmer starting at the 3′ end, with nine additional nucleotides (5′ GAA TAG CGA 3′) at the 5′ end and biotin at the 3′ end. The ligation probe is made of a phosphodiester oligo-DNA with a phosphate at the 5′ end and digoxigenin at the 3′ end. For 2′MOE-AO quantification, the template probe (5′ GAA TAG CGA ACC TTC CGA CGC TGT CTA GG 3' (BIO)) and the ligation probe (5′ TCG CTA TTC 3' (DIG)) were synthesized by Integrated DNA Technologies (IDT).

To prepare the muscle samples, tissues were powdered in liquid nitrogen and lysed in cooled RIPA buffer, followed by sonication. Protein concentration was determined using a BCA assay. For the quantification, 170 μL of protein lysate (0.03 μg/μL) was combined with the same volume of 0.05 μM template probe in hybridization buffer and incubated at 37°C for 1 h. The mixture was then transferred to Pierce NeutrAvidin Coated Plates (Thermo Fisher, #15217), with 150 μL per well in duplicate, and incubated at 37°C for 30 min.

After three washes with washing buffer, ligation was performed at room temperature for 2 h in the presence of 0.067 μM ligation probe, 400 U/mL T4 ligase, and 0.05 mM ATP in 1X One-Phor-All Plus buffer. Following antigen-antibody reaction, anti-digoxigenin antibody conjugated to ALP (Roche, #11093274910) was used at a 1:2000 dilution in 150 μL of SuperBlock (TBS) blocking buffer (Thermo Fisher, #37581) for 30 min at room temperature. After three additional washes, 150 μL of AttoPhos Substrate was added to each well, and the plate was incubated at 37°C for 20 min. Fluorescence was then measured using a microplate reader (Tecan Spark), with excitation set to 450/50 nm and emission set to 580/50 nm.

### Western blot analysis

Sample preparation: pre-frozen quadriceps muscle powered in liquid nitrogen and lysed in RIPA buffer with 1x Halt proteinase inhibitor cocktail (ThermoFisher Scientific, #78430). Protein concentration measured by BCA assay (ThermoFisher Scientific, Pierce BCA protein assay kits, #A55864).

Fifty μg protein and positive DUX4 protein control 2ul (myoblast lysate which transfected *DUX4* gene) and 5 μl ladder were separated by SDS-PAGE on any KD Mini-PROTEAN TGX Stain-Free protein gel (Bio-Rad; 200 V, 30 min) and subsequently transferred onto nitrocellulose membranes by semi-dry transfer (Trans-Blot Turbo Transfer System, Bio-Rad) with 10 min. Visualization of total protein in the membrane was taken using stain-free image of blot under ChemiDoc Imaging Systems (Bio-Rad). This will be used for specific protein normalization. Then, the membranes were blocked with 5% non-fat milk in Tris-buffered saline containing 0.1% Tween 20 (TBST) incubated for 1 h at room temperature, followed primary antibody incubation, DUX4 monoclonal antibody (P4H2) (1:500, ThermoFisher Scientific, # MA5-16147) at 37°C for 90 min. The membranes were washed three times with 0.1% TBST followed by incubation with horseradish peroxidase-conjugated corresponding second antibodies goat-mouse (1:5000, Santa Cruz) for 1 h at room temperature, after three time washing with 0.1% TBST, the signal was detected using SuperSignal West Femto Substrate (ThermoFisher Scientific) and imaged by ChemiDoc MP Imaging Systems (Bio-Rad). Western blot quantification analysis used Image Lab software (v5.12), protein normalization was used by stain-free imaging technology (Bio-Rad).

### Statistical analysis

Statistical analyses were conducted using GraphPad Prism version 10 (GraphPad Software, San Diego, CA, USA). One-way analysis of variance (ANOVA) was applied to compare differences among multiple groups, followed by Tukey’s multiple comparisons test for post hoc pairwise analyses. A significance threshold of *p* < 0.05 was considered statistically significant. Reported *p* values represent results from Tukey’s post hoc tests when the overall one-way ANOVA indicated significance (*p* < 0.05); when the one-way ANOVA result was not significant (*p* > 0.05), this was explicitly stated.

Comparisons of grip strength measurements between mid-point and endpoint assessments were analyzed using a two-way repeated-measures ANOVA followed by Tukey’s multiple comparisons test, with *p* < 0.05 considered significant. Correlation analyses were performed using Pearson’s correlation coefficient (r), and statistical significance was defined as *p* < 0.05. All data are presented as mean ± standard deviation (SD).

## Data availability

The authors confirm that the data supporting the findings of this study are available within the article and its supplemental information. The datasets used and/or analyzed during the current study are available from the corresponding author (Y.-W.C.) upon request.

## Acknowledgments

The study is supported by 10.13039/100009824FSH Society and 10.13039/100005202Muscular Dystrophy Association. Y.-W.C., A.Z., and Z.C. are partially supported by SOLVE FSHD/FSHD Canada foundation, 10.13039/100000002NIH/NICHD 1R21HD103993 or 10.13039/100000002NIH/10.13039/100000069NIAMS 1R21AR080887.

## Author contributions

Y.-W.C. and T.Y. contributed to the idea of the study; Y.-W.C. and A.Z. designed the experiments; A.Z., K.R.Q.L., and Z.C. conducted experiments. A.Z. and Z.C. analyzed the data. Y.-W.C. and A.Z. wrote the manuscript. All authors read, edit, and approved of the final manuscript.

## Declaration of interests

Y.-W.C. and T.Y. are co-inventors of the antisense oligonucleotides evaluated in the study. US 16649122, Europe 18859092; Canada 3099522. T.Y. is a Co-founder and shareholder of OligomicsTx, Inc.

## Supplemental information

Document S1. Figures S1–S4 and Tables S1–S3

Document S2. Article plus supplemental information
