# Supplementary material for: DUX4 reduction and muscle function improvement by subcutaneous delivery of gapmer antisense oligonucleotides
Source: Mol Ther Nucleic Acids. 2025 Dec 8;37(1):102791. doi: 10.1016/j.omtn.2025.102791 (PMC12795667; doi:10.1016/j.omtn.2025.102791)
Supplement: Document S1. Figures S1–S4 and Tables S1–S3 [file mmc1.pdf]

OMTN, Volume *37*

Supplemental information

DUX4 reduction and muscle function improvement by subcutaneous delivery of gapmer antisense oligonucleotides

Aiping Zhang, Kenji Rowel Q. Lim, Ze Chen, Toshifumi Yokota, and Yi-Wen Chen

Figure S1

A

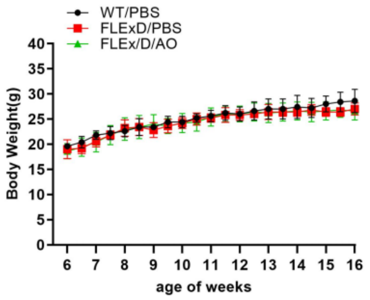

B

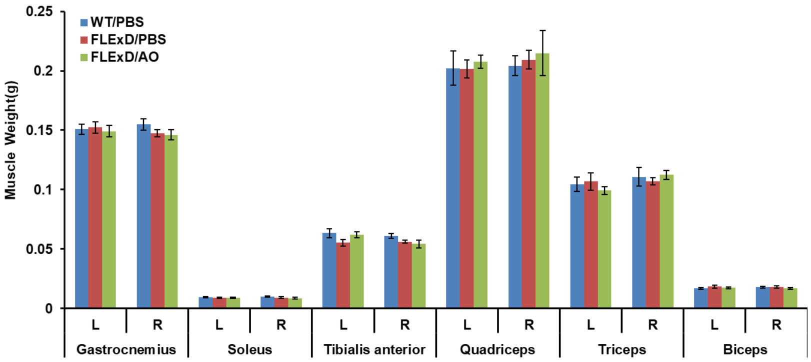

Figure S1. Treatment of 2′MOE gapmer did not change body weight or muscle weight significantly in FLExDUX4 Mice. A. body weight changes during the10-week trial. B. Individual muscle weight of the three groups of mice. WT/PBS, wild-type siblings treated with vehicle (PBS); FLExD/PBS, FLExDUX4 mice treated with vehicle (PBS); FLExD/AO, FLExDUX4 mice treated with 2′MOE-AO. All data are presented as mean (SD), n=5 per group.

Figure S2

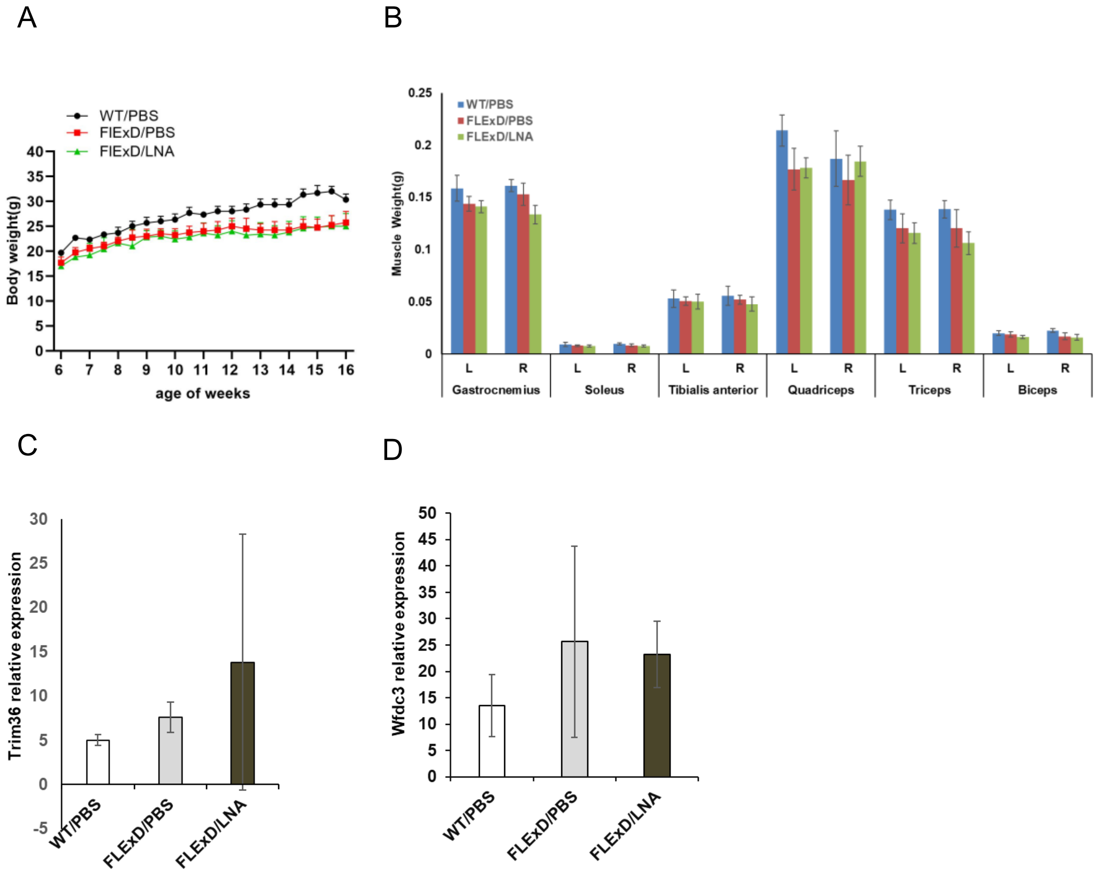

Figure S2. Treatment of LNA gapmer did not change body weight or muscle weight significantly in FLExDUX4 Mice. A. body weight changes during the10-week trial. B. Individual muscle weight of the three groups of mice. C. Expression of trim36. D. Expression of wfdc3. WT/PBS, wild-type siblings treated with vehicle (PBS), n=4; FLExD/PBS, FLExDUX4 mice treated with PBS, n=5; FLExD/LNA, FLExDUX4 mice treated with LNA-AO, n=5. All data are presented as mean (SD).

Figure S3

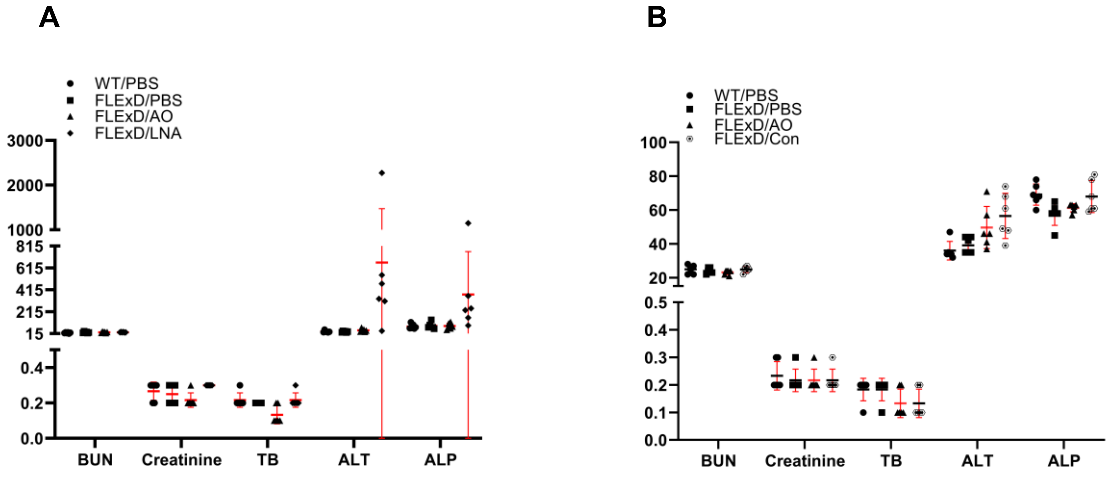

Figure S3. Serum biochemistry panel of liver and kidney listed in the graph. BUN, blood urea nitrogen (mg/dl); ALT, Alanine transaminase (U/L); ALP, alkaline phosphatase (U/L); TB, total bilirubin (mg/dl). All data are presented as mean (SD). A. indicated serum biochemistry level from10-week treatment trial of 2'MOE-AO or LNA-AO on FLExDUX4 mice. B. indicated serum biochemistry level from repeated 2'MOE-AO or 2'MOE-Con 10-week trial on FLExDUX4 mice

Figure S4

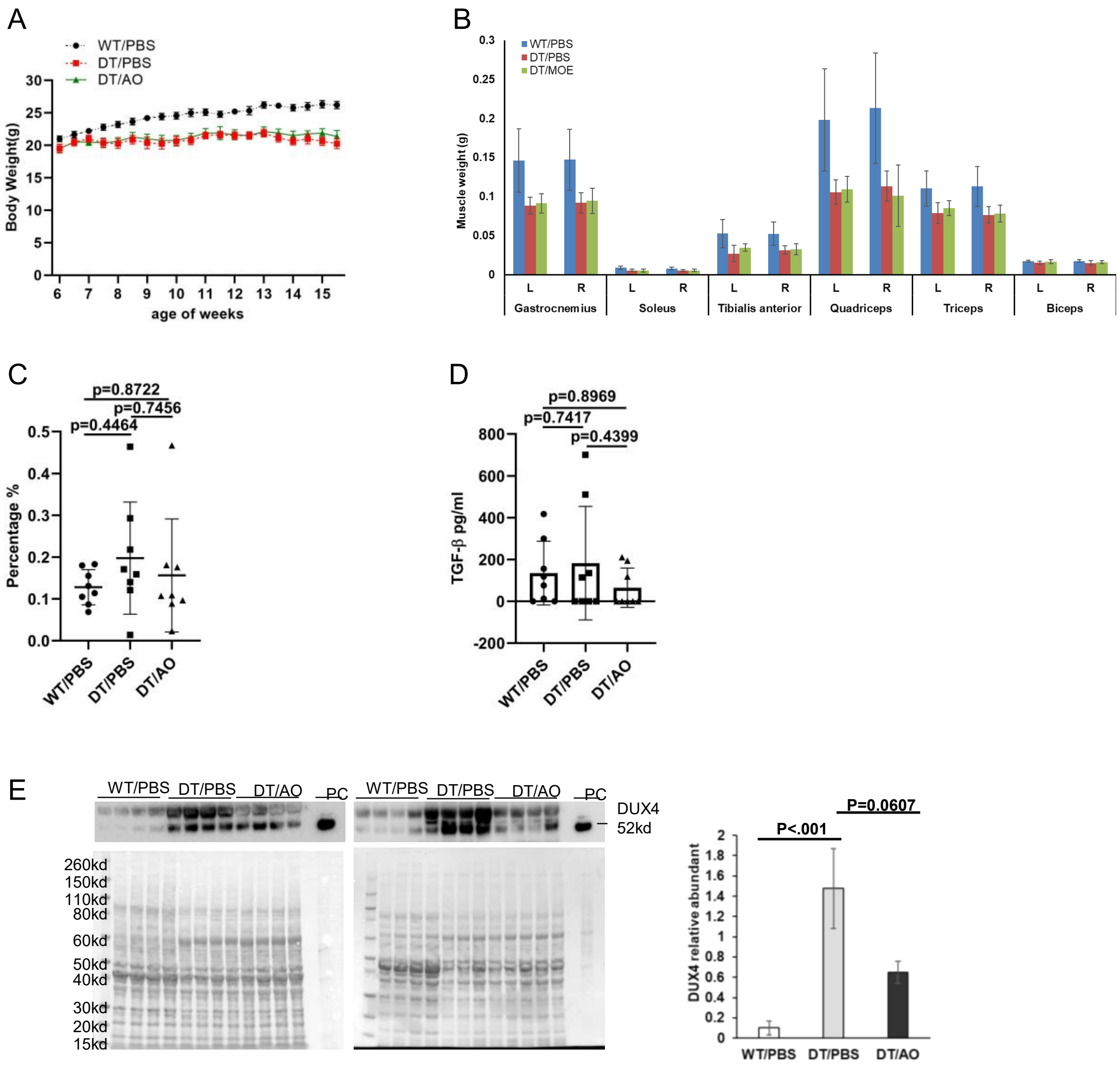

Figure S4. 2′MOE gapmer treatment did not significantly affect body or muscle weight, fibrosis, serum TGF-β1, or DUX4 protein in DT mice. A. Body weight over the 10-week treatment period. B. Muscle weights in each group. C. Percentage of fibrosis. D. Serum TGF-β1 level. E. DUX4 protein levels in quadriceps (Western blot). Human myoblasts transfected with DUX4 were used as a positive control (PC). Protein loading was normalized; DUX4 antibody used P4H2. WT/PBS, *ACTA1-MCM* Cre-positive wild-type littermates treated with PBS; DT/PBS, *ACTA1.MCM;FLExDUX4* mice treated with PBS; DT/AO, *ACTA1.MCM;FLExDUX4* mice treated with 2′MOE-AO. All data are presented as mean (SD), n = 8 per group. one-way ANOVA with Tukey's multiple comparisons test, $p < 0.05$ as significant.

Table S1 Overview antisense oligonucleotides targeting DUX4 mRNA

Chemistry, backbone	Conjugates	Target location	DUX4 reduction	Delivery	Mechanisms	References
2′-OMe, PS	none	Exon 2, Exon 3, SA, PAS	30~50%	in vitro	steric blocking	Vanderplanck et al. 2011[1]
PMO	none	Exon 2, Exon 3, PAS	~100%	in vitro	steric blocking	Chen et al. 2016[2]
			~80%	im		
PMO	none	Exon 3, SC, PAS	~40-50%	in vitro	steric blocking	Marsollier et al. 2016[3]
2′-OMe, PS	none	Exon 2, Exon 3, SA	~90%	in vitro	steric blocking	Ansseau et al.2017[4]
PMO	octa-guanidinium dendrimer	Exon 3, SA	~30-fold decrease	im		
PMO	octa-guanidine dendrimer	Exon 3, SA	Not	im	steric blocking	Derenne et al. 2020[5]
LNA gapmer, PS	none	Exon 1, Exon 3	~100%	in vitro	RNase H-mediated cleavage	Lim et al. 2020[6]
			~84%	i.m		
2’MOE gapmer, PS	none	Exon 3	~100%	in vitro	RNase H-mediated cleavage	Lim et al. 2021[7]
			~65%	im		
PMO	octaguanidine dendrimer	Exon 3, CS3, PAS	50%	ip	steric blocking	Lu-Nguyen et al. 2021[8]
cET gapmer	Palmitoyl	Exon 1	~37%	s.c.	RNase H-mediated cleavage	Bouwman et al. 2021[9]
PMO	Chitosan-shelled NBs	Exon 3, CS	0%	in vitro	steric blocking	Falzarano et al., 2021[10]
PMO	octaguanidine dendrimer	Exon 3, CS, PAS	~40%	ip	PAS and CS3	Lu-Nguyen et al. 2022a[11]
PMO	octaguanidine dendrimer	Exon 3, CS, PAS	60%	ip	PAS and CS3	Lu-Nguyen et al. 2022b[12]
ALNA[Ms] gapmer, PS	none	Exon 3	~35 to 50%	s.c	RNase H-mediated cleavage	Kakimoto et al. 2023[13]

Abbreviations: 2′-OMe, 2′-O-methyl; PS, phosphorothioated; SA, splice acceptor; PMO, phosphorodiamidate morpholino oligomer; PAS, polyadenylation signal; LNA, locked nucleic acid; 2′MOE, 2′-O-methoxyethyl; cEt, constrained ethyl; ALNA[MS], 2′-N-methanesulfonyl-2′-amino-locked nucleic acid; CS, cleavage site; im, intramuscular Injection; s.c., subcutaneous injection; ip, intraperitoneal injection.

Table S2. Overview antisense oligonucleotides animal therapeutic trial by system delivery

Animal Model	Tamoxifen dosage (IP)	Interval Tamoxifen and AO	Chemistry	conjugate	regimen	Duration	DUX4 reduction %	Toxicity	Reference
ACTA1-MCM;FLExDUX4	N/A		cEt gamper, PS	Palmitoyl	50mg/kg, s.c., 2/wk,	3 weeks	37%	No liver or kidney toxicity	Bouwman et al. 2021[9]
					50mg/kg, s.c., 2/wk 4 doses plus 50mg/kg/wk 6 doses	10 weeks	40%		
	2.5mg/k, 2/wk	Day 2 after first TMX	PMO	octa-guanidinium dendrimer	10mg/kg, ip,	30 days	50%	N/A	Lu-Nguyen et al. 2021[8]
	1.5mg/k, 2/wk	1 week after 2nd TMX	PMO	octa-guanidinium dendrimer	10mg/kg, 2/wk, ip, 12 doses	8 weeks	40 to 60%	N/A	Lu-Nguyen et al. 2022b[12]
	2.5mg/k, 2/wk	Day 2 after first TMX	PMO	octa-guanidinium dendrimer	10mg/kg, ip on day2,8,16 and 22 after first TMX	22 days	~50%	N/A	Lu-Nguyen et al. 2022a[11]
	N/A		ALNA[Ms] gapmer, PS		10 or 15mg/kg, s.c., Q2W	10 weeks	40%	No liver or kidney injury	Kakimoto et al. 2023[13]
	5mg/kg	One dose 17 days before the endpoint			15 or 30mg/kg, s.c., Q2W	6 weeks	45%		
	7.5mg/kg	One dose 14 days before the endpoint			10 mg/kg, s.c., Q2W	10 weeks	~35%		

Abbreviations: Wk, week; 2/wk, twice a week; Q2W, dosing interval of two weeks TMX, Tamoxifen; s.c., subcutaneous injection; ip: intraperitoneal injection. 2′-OMe, 2′-O-methyl; PS, phosphorothioated; PMO, phosphorodiamidate morpholino oligomer; cEt, constrained ethyl; 5′-mC, 5′-methylcytosines; ALNA[MS], 2′-N-methanesulfonyl-2′-amino-locked nucleic acid.

Table S3. Summary of DUX4 reduction by our antisense oligonucleotides in systemic therapeutic trial on animal models

Animal	TMX dosage (ip)	Interval between TMX and AO first dose	Chemistry, backbone	Regimen	Duration	DUX4 reduction % (mean $_{untreated}$ – mean $_{AO\text{-}treated}$) / mean $_{untreated}$					Liver and kidney toxicity (chemistry of serum test)
						Quadriceps	Triceps	Tibialis anterior	Biceps	Gastrocnemius	
FLExDUX4	N/A	N/A	LNA gapmer, PS	20 mg/kg, s.c, 2/wk, 21 doses	10 weeks	60% (19.5-6.4)/19.5					ALP and ALT increased
	N/A	N/A	2'MOE gapmer, PS		10 weeks		55.0% (33.7-15.2)/33.7				20mg/kg for 10 weeks trial didn't observe liver and kidney toxicity in both 2'MOE-AO and 2'MOE-control
					10 weeks	59.7% (PBS treated) (6.0-2.4)/6.0 or 61.3% (Scramble treated) (6.2-2.4)/6.2	53.9% (PBS treated) (56.1-25.8)/56.1 or 55.9% (Scramble treated) (58.7-25.8)/58.7	83.6% (PBS treated) (32.8-5.4)/32.8 or 81.6% (Scramble treated) (29.3-5.4)/29.3	66.5% (PBS treated) (18.8-6.3)/18.8 or 59.4% (Scramble treated) (15.5-6.3)/15.5	59.4% (PBS treated) (33.5-13.6)/33.5 or 60.7% (Scramble treated) (34.6-13.6)/34.6	
				5mg/kg, 9 doses	4 weeks	44% (10.0-8.2)/10	67.7% (10.0-3.2)/10.0				
				20mg/kg, 9 doses	4 weeks	57.7% (10.0-4.2)/10	80.0% (10.0-2.0)/10.0				
				50mg/kg, 9 doses	4 weeks	72.6% (10.0-2.7)/10	88.5% (10.0-1.2)/10.0				N/A
ACTA1-MCM;FLExDUX4	5mg/kg, one dose	before 36 hours AO administration		20 mg/kg, s.c., every other day, 6 doses	2 weeks		66.5% (191.0-63.9)/191.0				N/A
	5mg/kg, 2/wk, 5 doses			20 mg/kg, 2/wk, 21 doses	10 weeks		54.4% (76.0-34.7)/76.0				

Abbreviations: 2/wk, twice a week; TMX, Tamoxifen; s.c, subcutaneous injection; ip, intraperitoneal injection; LNA, locked nucleic acid; 2′MOE, 2′-O-methoxyethyl; PS, phosphorothioated; ALP, alkaline phosphatase; ALT, Alanine aminotransferase; Untreated, include PBS treated and scramble treated;

Reference

1. Vanderplanck, C., Ansseau, E., Charron, S., Stricwant, N., Tassin, A., Laoudj-Chenivesse, D., Wilton, S. D., Coppee, F., and Belayew, A. (2011). The FSHD atrophic myotube phenotype is caused by DUX4 expression. *PLoS One* **6**: e26820.
2. Chen, J. C., King, O. D., Zhang, Y., Clayton, N. P., Spencer, C., Wentworth, B. M., Emerson, C. P., Jr., and Wagner, K. R. (2016). Morpholino-mediated Knockdown of DUX4 Toward Facioscapulohumeral Muscular Dystrophy Therapeutics. *Mol Ther* **24**: 1405-1411.
3. Marsollier, A. C., Ciszewski, L., Mariot, V., Popplewell, L., Voit, T., Dickson, G., and Dumonceaux, J. (2016). Antisense targeting of 3' end elements involved in DUX4 mRNA processing is an efficient therapeutic strategy for facioscapulohumeral dystrophy: a new gene-silencing approach. *Hum Mol Genet* **25**: 1468-1478.
4. Ansseau, E., Eidahl, J. O., Lancelot, C., Tassin, A., Matteotti, C., Yip, C., Liu, J., Leroy, B., Hubeau, C., Gerbaux, C., *et al.* (2016). Homologous Transcription Factors DUX4 and DUX4c Associate with Cytoplasmic Proteins during Muscle Differentiation. *PLoS One* **11**: e0146893.
5. Derenne, A., Tassin, A., Nguyen, T. H., De Roeck, E., Jenart, V., Ansseau, E., Belayew, A., Coppee, F., Decleves, A. E., and Legrand, A. (2020). Induction of a local muscular dystrophy using electroporation in vivo: an easy tool for screening therapeutics. *Sci Rep* **10**: 11301.
6. Lim, K. R. Q., Maruyama, R., Echigoya, Y., Nguyen, Q., Zhang, A., Khawaja, H., Sen Chandra, S., Jones, T., Jones, P., Chen, Y. W., *et al.* (2020). Inhibition of DUX4 expression with antisense LNA gapmers as a therapy for facioscapulohumeral muscular dystrophy. *Proc Natl Acad Sci U S A* **117**: 16509-16515.
7. Lim, K. R. Q., Bittel, A., Maruyama, R., Echigoya, Y., Nguyen, Q., Huang, Y., Dzierlega, K., Zhang, A., Chen, Y. W., and Yokota, T. (2021). DUX4 Transcript Knockdown with Antisense 2'-O-Methoxyethyl Gapmers for the Treatment of Facioscapulohumeral Muscular Dystrophy. *Mol Ther* **29**: 848-858.
8. Lu-Nguyen, N., Malerba, A., Herath, S., Dickson, G., and Popplewell, L. (2021). Systemic antisense therapeutics inhibiting DUX4 expression ameliorates FSHD-like pathology in an FSHD mouse model. *Hum Mol Genet* **30**: 1398-1412.
9. Bouwman, L. F., den Hamer, B., van den Heuvel, A., Franken, M., Jackson, M., Dwyer, C. A., Tapscott, S. J., Rigo, F., van der Maarel, S. M., and de Greef, J. C. (2021). Systemic delivery of a DUX4-targeting antisense oligonucleotide to treat facioscapulohumeral muscular dystrophy. *Mol Ther Nucleic Acids* **26**: 813-827.
10. Falzarano, M. S., Argenziano, M., Marsollier, A. C., Mariot, V., Rossi, D., Selvatici, R., Dumonceaux, J., Cavalli, R., and Ferlini, A. (2021). Chitosan-Shelled Nanobubbles Irreversibly Encapsulate Morpholino Conjugate Antisense Oligonucleotides and Are Ineffective for Phosphorodiamidate Morpholino-Mediated Gene Silencing of DUX4. *Nucleic Acid Ther* **31**: 201-207.
11. Lu-Nguyen, N., Malerba, A., Antoni Pineda, M., Dickson, G., and Popplewell, L. (2022). Improving Molecular and Histopathology in Diaphragm Muscle of the Double Transgenic ACTA1-MCM/FLExDUX4 Mouse Model of FSHD with Systemic Antisense Therapy. *Hum Gene Ther* **33**: 923-935.
12. Lu-Nguyen, N., Dickson, G., Malerba, A., and Popplewell, L. (2022). Long-Term Systemic Treatment of a Mouse Model Displaying Chronic FSHD-like Pathology with Antisense Therapeutics That Inhibit DUX4 Expression. *Biomedicines* **10**.
13. Kakimoto, T., Ogasawara, A., Ishikawa, K., Kurita, T., Yoshida, K., Harada, S., Nonaka, T., Inoue, Y., Uchida, K., Tateoka, T., *et al.* (2023). A Systemically Administered Unconjugated Antisense Oligonucleotide Targeting DUX4 Improves Muscular Injury and Motor Function in FSHD Model Mice. *Biomedicines* **11**.
